# Offspring dependence on parental care and the role of parental transfer of oral fluids in burying beetles

**DOI:** 10.1186/s12983-018-0278-5

**Published:** 2018-08-29

**Authors:** Alexandra Capodeanu-Nägler, Madlen A. Prang, Stephen T. Trumbo, Heiko Vogel, Anne-Katrin Eggert, Scott K. Sakaluk, Sandra Steiger

**Affiliations:** 1 Institute of Evolutionary Ecology and Conservation Genomics, University of Ulm, Ulm, Germany; 2 Department of Evolutionary Animal Ecology, University of Bayreuth, Bayreuth, Germany; 3 Department of Ecology and Evolutionary Biology, University of Connecticut, Waterbury, CT USA; 4 Department of Entomology, Max-Planck-Institute for Chemical Ecology, 07745 Jena, Germany; 5 Behavior, Ecology, Evolution and Systematics Section, School of Biological Sciences, Illinois State University, Normal, IL 61790-4120 USA

**Keywords:** *Nicrophorus*, Burying beetles, Parental care, Trophallaxis, Starvation tolerance, Oral secretions, Offspring dependence

## Abstract

**Background:**

Immature stages of many animals can forage and feed on their own, whereas others depend on their parents’ assistance to obtain or process food. But how does such dependency evolve, and which offspring and parental traits are involved? Burying beetles (*Nicrophorus*) provide extensive biparental care, including food provisioning to their offspring. Interestingly, there is substantial variation in the reliance of offspring on post-hatching care among species. Here, we examine the proximate mechanisms underlying offspring dependence, focusing on the larvae of *N. orbicollis*, which are not able to survive in the absence of parents. We specifically asked whether the high offspring dependence is caused by (1) a low starvation tolerance, (2) a low ability to self-feed or (3) the need to obtain parental oral fluids. Finally, we determined how much care (i.e. duration of care) they require to be able to survive.

**Results:**

We demonstrate that *N. orbicollis* larvae are not characterized by a lower starvation tolerance than larvae of the more independent species. Hatchlings of *N. orbicollis* are generally able to self-feed, but the efficiency depends on the kind of food presented and differs from the more independent species. Further, we show that even when providing highly dependent *N. orbicollis* larvae with easy ingestible liquefied mice carrion, only few of them survived to pupation. However, adding parental oral fluids significantly increased their survival rate. Finally, we demonstrate that survival and growth of dependent *N. orbicollis* larvae is increased greatly by only a few hours of parental care.

**Conclusions:**

Considering the fact that larvae of other burying beetle species are able to survive in the absence of care, the high dependence of *N. orbicollis* larvae is puzzling. Even though they have not lost the ability to self-feed, an easily digestible, liquefied carrion meal is not sufficient to ensure their survival. However, our results indicate that the transfer of parental oral fluids is an essential component of care. In the majority of mammals, offspring rely on the exchange of fluids (i.e. milk) to survive, and our findings suggest that even in subsocial insects, such as burying beetles, parental fluids can significantly affect offspring survival.

## Background

Most animals eat to acquire nutrients that are essential to fulfil the energetic needs for their growth or reproduction [1, 2]. Generally, only a part of the food an individual consumes is absorbed, metabolised and converted into usable energy or nutrients, i.e. “digestive efficiency” [3–6]. Digestive efficiency reflects how effectively an individual can exploit food resources, and this might vary between species due to different capabilities to process and absorb food when the resource is very challenging, or due to different physiological requirements, irrespective of the type of food [3]. To overcome these challenges, parents in various animal taxa have evolved traits to provision offspring with food or to assist them with digestion. In mammals, for example, parental care is obligate. Females provide milk [7, 8], which is not only rich in lipids and proteins, but also contains bioactive components such as growth factors, hormones, or immunological factors that contribute to the development and protection of the young [9]. In pigeons, parents produce and feed their chicks ‘crop milk’ that contains nutrients, minerals and growth factors [10], as well as immune-active compounds such as immunoglobulins [11] and carotenoids [12]. Thus, food provisioning not only entails the breakdown and pre-digestion of food, but also the transfer of important ancillary compounds.

Unlike mammals or birds, parental food provisioning occurs in only about 1% of insect species [13]. Researchers have repeatedly suggested that when food is ephemeral (e.g., carrion, dung) or difficult to process (e.g., wood), insects are more likely to evolve some form of parental care, such as facilitating feeding of offspring, or protecting both the resource and the offspring from competitors, predators, or parasites [14, 15]. Wood roaches of the genus *Salganea*, for example, have evolved morphological adaptations of the mouthparts to facilitate the uptake of stomodeal substances via trophallaxis by the mother [16–18]. However, it is currently unclear whether these substances contain wood fragments, nutrients, and/or enzymes or other chemicals involved in the degradation of cellulose [19]. Generally, oral fluids exchanged by trophallaxis may include proteins that are regulators of growth, development, and behavioural maturation [20, 21]. Alternatively, parents might pass symbionts to offspring that are essential for their survival and growth. Altricial neonates of the wood-feeding cockroach *Cryptocercus punctulatus*, for example, cannot directly process wood, but instead rely on the hindgut fluids of their parents to acquire symbionts that are necessary for digestion (i.e. proctodeal trophallaxis) [22].

In contrast to wood or foliage, carrion is a highly nutritious and ephemeral resource that is easily digested [14]. Dipteran females are usually the first insects to arrive at a carcass and to deposit their eggs or first-instar larvae directly on top of the carrion, often in natural body openings or at wound sites [23]. The maggots then, without any parental assistance, immediately start feeding on the carrion at the site where they emerge. Most beetles in the family Silphidae also have larvae that depend on carrion as their food, and in all genera except *Nicrophorus*, the larvae feed independently. Only in *Nicrophorus* do adult beetles bury carcasses in an apparent attempt to monopolize and defend them for their young. In addition, parents provide food to their offspring and, within this genus, there is significant variation in the dependence of offspring on post-hatching parental care, most likely on parental feeding [24, 25]. The larvae of some species can easily feed and survive on a carcass without parental help, whereas others cannot. However, the evolutionary causes driving these differences in the dependence on parental care among species that utilize the same food resource remain obscure. One mathematical model for the evolution of parental care predicts that in species that provide care to their offspring, food provisioning is expected to evolve if it is more efficient than offspring self-feeding, or more efficient than parental efforts to guard against predators [26]. Also, the evolution of food provisioning promotes a mutual reinforcement between parental feeding and sibling competition, resulting in a unidirectional trend from no to full parental food provisioning [26]. Once parental feeding has evolved, coadaptation between parental and offspring traits may lead to the delayed ontogenetic development of traits that are necessary for offspring self-feeding [25].

However, to better understand which factors drive the evolutionary loss of independence, it is crucial to determine the proximate cause of offspring dependence. On what parental service do offspring actually rely, and which offspring traits differ between dependent and independent species? Are there insect hatchlings that have lost their ability to self-feed similar to neonates of altricial mammals and birds?

Here, we examine the proximate mechanisms underlying offspring dependence using burying beetles as a model system. Burying beetles are well-known for their habit of interring small vertebrate carcasses and providing extensive biparental care to their offspring before and after hatching [27–30]. In *N. vespilloides*, larvae are capable of self-feeding, but nevertheless beg for regurgitated pre-digested carrion from their parents [31, 32]. Parental regurgitations are hypothesized to ensure a sufficient food supply for larvae, when their mandibles are still soft and not fully sclerotized following larval moults [27]. In our previous study, we found that offspring of the three species *N. orbicollis*, *N. pustulatus*, and *N. vespilloides* show marked differences in their dependence on parental provisioning, or at least on post-hatching care [25]. *N. orbicollis*, which is one of the most basal species within the genus *Nicrophorus* [33], appears to be a beetle with obligatory parental care, as offspring do not survive in the absence of parents, whereas parental care is facultative in *N. pustulatus* and *N. vespilloides* [24, 25]. Likewise, parental care appears to be facultative in many other *Nicrophorus* species, including *N. mexicanus* [34], *N. defodiens, N. tomentosus* [24] and *N. quadripunctatus* [35]. This raises the question, therefore, as to why *N. orbicollis* is so exceptional among other *Nicrophorus* species with regard to offspring dependency, and more particularly, what causes the striking helplessness of offspring in the absence of parents. As our study aimed to investigate the proximate mechanisms of offspring dependency, we focused on the most dependent species studied to date, *N. orbicollis*, and drew comparisons to more independent species when required.

We first tested the hypothesis that larvae of the different species are equally efficient at self-feeding, but that *N. orbicollis* parents may invest fewer resources into eggs or their larvae may be fast metabolisers. Both scenarios would result in larvae with a higher food demand in a shorter time period. To test this hypothesis, we did not measure nutrient content in eggs nor the metabolic rate of larvae, but rather used starvation tolerance of larvae as a proxy for a high resource need per time unit. In addition, we noted larval mass at hatching as an indicator of egg investment. In the next step, we investigated whether *N. orbicollis* larvae are capable of self-feeding from the time of hatching, or whether the expression of morphological adaptations needed for self-feeding is delayed compared to more independent species. Additionally, we tested whether the self-feeding capacity of larval *N. orbicollis* is inferior to the self-feeding capacity of the more independent species. In burying beetles, parental food provisioning entails not only the regurgitation of partially digested carrion, but also the transfer of oral fluids, which might also contain important microbial symbionts, enzymes, or growth hormones (see e.g. [20]). Thus, upon discovering that larval *N. orbicollis* are particularly effective in feeding on pieces of baby mice, we tried to rear them on an easily digestible diet of liquefied carrion either supplemented with parental oral secretions or not. With this experiment, we also tested the hypothesis that the characteristics of larval mandibles play an important role in determining offspring dependence. In *N. vespilloides*, a previous study has shown that larvae that receive at least 12 h of parental care survive well, and average larval mass does not significantly increase with longer care [28]. In a final experiment, we therefore attempted to determine the minimum duration of post-hatching care required for larval survival to adulthood in *N. orbicollis*.

## Methods

### Origin and maintenance of experimental beetles

*N. vespilloides* used in the study were descendants of beetles collected from carrion-baited pitfall traps in a forest near Ulm, Germany (48°25′03″N, 9°57′45″E). Cultures of *N. pustulatus* and *N. orbicollis* were established at Ulm University from outbred colonies maintained at the Institute of Zoology, University of Freiburg, Germany. We maintained outbred colonies of both species by introducing beetles captured in baited pitfall traps established in a forested area near Lexington, Illinois, U.S.A. (40°39′57″N, 88°53′49″W). All beetles were held in temperature-controlled incubators at 20 °C on a 16:8 h light:dark cycle. Before the experiments, groups of up to five adult beetles of the same sex and family of each species were kept in small plastic containers (10 × 10 cm and 6 cm high) filled with moist peat. Beetles were fed freshly decapitated mealworms ad libitum twice a week. At the time of experiments, beetles were virgin and between 20 and 30 days of age.

### Experimental design

#### Experiment 1: Starvation tolerance of larvae

Larval *N. orbicollis* do not survive in the absence of post-hatching care [24, 25]. In this experiment, we measured starvation tolerance of *N. orbicollis* offspring in comparison with the more independent species, *N. pustulatus* and *N. vespilloides*. For this, we randomly selected non-sibling pairs of male and female beetles, placed them in small plastic containers filled with peat (10 × 10 cm and 6 cm high), and induced reproduction by providing them with a 20 g (± 3 g) thawed mouse carcass (Frostfutter.de – B.A.F Group GmbH, Germany). In the case of the nocturnal species, *N. orbicollis* and *N. pustulatus*, mice were provided during the dark portion of the photoperiod, whereas for crepuscular *N. vespilloides*, mice were provided during the light portion. To prevent hatching larvae from access to food, we transferred parents and the carcass to new boxes filled with peat after the egg-laying period (see [25]), and left the eggs to hatch in the old container. From the expected time of larval hatching, we checked for larvae every hour. Then, for each species, we set up a minimum number of 30 larvae from at least six different families (number of larvae, families: *N. orbicollis*: 36, 6; *N. pustulatus*: 33, 11; *N. vespilloides*: 39, 9) to avoid any family effect on larval survival. One larva each was added on top of a moistened paper tissue in a shallow plastic tray (3 × 3 cm × 0.5 cm high) without access to food, and kept in a temperature-controlled room at 20 °C. We then checked for the survival of each larva every hour up to a maximum of 42 h, and moistened the paper tissue, if necessary, to ensure an adequate supply of water to the larvae. Finally, we recorded the number of hours that larvae survived.

#### Experiment 2: Self-feeding ability on different food resources

Here, we set up non-sibling pairs of beetles as in experiment 1 for the three species, *N. orbicollis*, *N. pustulatus*, and *N. vespilloides*. As before, parents and their carcass were transferred to new boxes after the egg laying period, and the old boxes were checked at least every 8 h for the hatching of larvae. To measure interspecific variation in the ability of larvae to self-feed when parents are absent, we established three treatments per species (*n* = 15 for each species and treatment) in which we offered individual larvae one of three different food resources ranging from very challenging food to very easily accessible and digestible food: (1) carrion prepared by parents in the pre-hatching period without a hole created by parents; (2) carrion prepared by parents in the pre-hatching period with a hole in the carcass created by parents; (3) small pieces of baby mice. Generally, burying beetle parents create an opening in the carcass shortly before or after larval hatching, allowing larvae direct access to the food [28]. To ensure that we obtained approximately equal numbers of prepared carcasses with (*n* = 15) and without a hole (*n* = 15), we set up additional pairs for reproduction in each species. Thus, for treatments 1 and 2, we provided 50 pairs in each species with a 20 g (± 3 g) thawed mouse carcass and allowed them to provide pre-hatching care according to their species-specific duration (*N. orbicollis*: 120 h; *N. pustulatus*: 80 h; *N. vespilloides*: 70 h). We then inspected prepared carcasses for an opening in the integument and assigned them to the treatment “prepared carrion without hole” in those instances where there was no hole. Carcasses that had already been processed and opened by the parents were additionally cut open using scissors and assigned to the treatment “prepared carrion with hole”. All food resources were offered in small plastic containers without peat (10 × 10 cm and 6 cm high), but lined with moist paper tissue. As soon as the larvae hatched, their initial mass (0 h) was determined to 0.01 mg using a precision scale (Kern ABT 220-5DM, Kern und Sohn GmbH, Balingen, Germany) before allowing them access to a food resource. Immediately thereafter, one larva each was randomly added on top of one of the three food resources. Larvae were then weighed again 2 h later to detect any changes in larval mass during this time interval.

#### Experiment 3: Effect of oral secretions on larval *N. orbicollis*

Here, we determined whether larvae of the most dependent species, *N. orbicollis*, could be reared in the absence of their parents when provided with a liquidized paste of baby mice mixed with or without oral fluids of their parents. For this, we established 40 pairs of male and female beetles, 20 of which were set up 2 days in advance and used for the extraction of oral secretions. The other 20 pairs served to provide larvae for the actual experiment. As before, parents and the carcass were transferred to new boxes after the egg-laying period, leaving the eggs in the old boxes to hatch. We established two treatment groups in which we provided larvae with (1) a paste of baby mice that included oral secretions of care-giving male or female parents that had been given access to larvae and a carcass for 24–48 h (*n* = 35), or (2) a paste of baby mice without oral secretions of parents (*n* = 35). To prepare the paste of baby mice, we placed 30 dead and frozen baby mice (1–3 g; Frostfutter.de—B.A.F Group GmbH, Germany) into a blender together with 30 mL of water, and mixed them until the paste was homogenous. To obtain the regurgitated oral fluids from a parent, we gently squeezed the thoracic-abdominal region of a beetle with a pair of forceps and collected the secretions with a pipette. For the experiments, we placed 5 larvae from one family (*n* = 7 for each treatment) that had hatched at the same time together in a petri dish containing a moist paper tissue. We then checked for the survival of larvae three times a day, and exchanged both the moist paper tissue and the food when larvae were still alive. We recorded the number of hours each larva survived.

In the group including oral secretions of parents (1), larvae were provided with 5 μL of oral secretions directly added on top of the moist paper tissue for the first 24 h. In addition, we added two 0.2 mL Eppendorf tubes containing 5 mg of baby mouse paste mixed with 5 μL of oral secretions of a parental beetle. Oral secretions were always freshly obtained from the parental beetles. In the group without oral secretions (2), larvae in the first 24 h were only provided with two 0.2 mL Eppendorf tubes that contained approximately 5 mg of baby mouse paste. The Eppendorf tubes in both treatments were sliced open at both ends to facilitate ready access of larvae to the food. After 24 h, larvae of both treatments received one 0.5 mL Eppendorf tube containing baby mouse paste without oral secretions. After 48 h, larvae received one opened baby mouse carcass and one 0.5 mL Eppendorf tube containing baby mouse paste without oral secretions. As parental regurgitations in *N. orbicollis* substantially decrease after 48 h, and larval survival and mass are subsequently not reduced in the absence of care ([36, 37], see also experiment 4), we opted to provide larvae solely with mouse carcasses thereafter. After 120 h, surviving larvae were placed into boxes with soil and provided with two opened baby mouse carcasses in succession, the first of which was left for 8 h, after which it was exchanged with the second carcass for an additional 8 h. This was done to ensure a sufficient food supply for larvae just prior to pupation. After the second carcass was removed, the larvae were left to pupate.

#### Experiment 4: Duration of post-hatching care needed to ensure development of larval *N. orbicollis*

The aim of this experiment was to determine the minimum duration of post-hatching care needed to ensure survival of larvae. To test this, we set up 200 non-sibling pairs of *N. orbicollis* beetles as in the previous experiments. After the egg laying period (see [25]), parents and their carcass were transferred to new boxes, and the old boxes were checked every 8 h for the hatching of larvae. To control for variation between families and individual differences in behaviour [38], we provided each pair of beetles with a brood of 15 newly hatched larvae of mixed parentage ([25, 39], see also [40]) Burying beetles exhibit temporally-based kin discrimination in which they kill any larvae arriving on the carcass before their own eggs would have hatched [41]. Hence, we only provided pairs with larvae after their own larvae had begun hatching. The larvae were placed directly onto the carcass, in which we had cut a hole through the skin earlier to facilitate larval access to the carrion in each of the treatments. We then allowed parents to provide post-hatching care for 1 h, 3 h, 6 h, 12 h, 24 h, or 48 h (*n* = 15 or 16 per treatment). In addition, we established a “pre-hatching care” treatment (*n* = 15), in which parents were only allowed to prepare the carcass, but were prevented from providing post-hatching care (“0 h”). Finally, we also established a “full-care” treatment, in which parents were allowed to prepare the carcass and to provide post-hatching care until larvae dispersed (8 ± 2 days). As soon as the surviving larvae of each brood left the carcass for pupation, they were counted and weighed.

### Statistical analysis

All data were analysed and plotted using R version 3.1.2 (R Core Team 2014) or SPSS version 21.0 (Chicago, IL, USA). For experiments 1 and 3, we used the Kaplan-Meier method in SPSS to estimate survival of larvae as a function of time. To test for differences in larval survival between the three species in experiment 1 and the two treatments in experiment 3, we used a log-rank test in SPSS. For experiment 2, we used the relative change in larval mass between 0 h and 2 h as a proxy to assess the ability to self-feed in each species. As larval mass at hatching differed among species (GLM with Gaussian errors: *F*_2,177_ = 517.69, *P* < 0.001), we first divided the absolute change in larval mass by the mass of each larva at hatching. We then applied generalised linear models (GLMs) with Gaussian distribution with species, treatment and species*treatment as fixed factors and the relative change in larval mass as the dependent variable. To identify species-specific treatment effects, we continued with GLMs followed by pairwise comparisons with Bonferroni correction for multiple testing within each of the three species in which treatment was included as a fixed factor and the relative change in larval mass as the dependent variable. For experiment 4, we included duration of post-hatching care (0 h, 1 h, 3 h, 6 h, 12 h, 24 h, 48 h, full care) as a fixed factor, and the absolute number of larvae that survived and mean larval mass per brood as dependent variables. We then applied GLMs with Poisson distribution followed by pairwise comparisons with Bonferroni correction for multiple testing. In addition, we compared the mean larval mass per brood at dispersal by using a GLM with Gaussian distribution.

## Results

### Experiment 1: Starvation tolerance of larvae

**Fig. 1:**
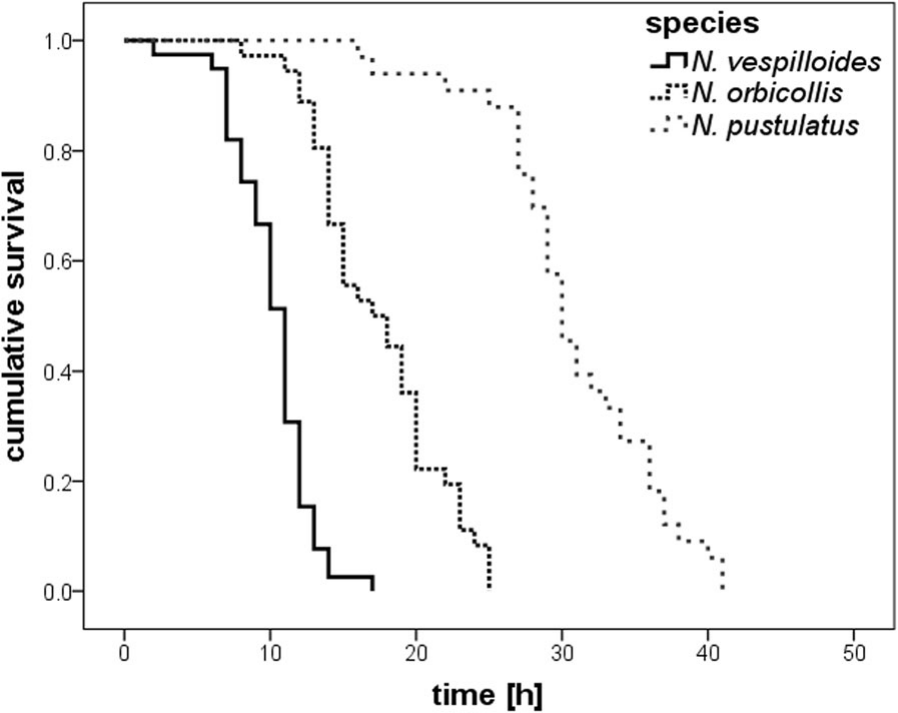
Survival of larvae without access to food in *N. orbicollis* (*N* = 36), *N. pustulatus* (*N* = 33), and *N. vespilloides* (*N* = 39) [h]. Kaplan-Meier estimated survival curves

Survival of larvae without access to food varied significantly among the three species (log-rank test, for all: *P* < 0.001, see Fig. 1). On average, highly dependent *N. orbicollis* larvae survived longer (mean: 17.44 ± SE 0.75 h) than larval *N. vespilloides* (mean: 10.21 ± SE 0.43 h) which show an intermediate dependence on parental care (log-rank test, *x*^*2*^ = 54. 28, *P* < 0.001). However, *N. orbicollis* larvae survived significantly shorter than the highly independent *N. pustulatus* larvae (mean: 30.82 ± SE 1.04 h) (log-rank test, *x*^*2*^ = 62.53, *P* < 0.001). *N pustulatus* larvae also survived longer than larval *N. vespilloides* (log-rank test, *x*^*2*^ = 77.03, *P* < 0.001).

### Experiment 2: Self-feeding ability on different food resources

**Fig. 2:**
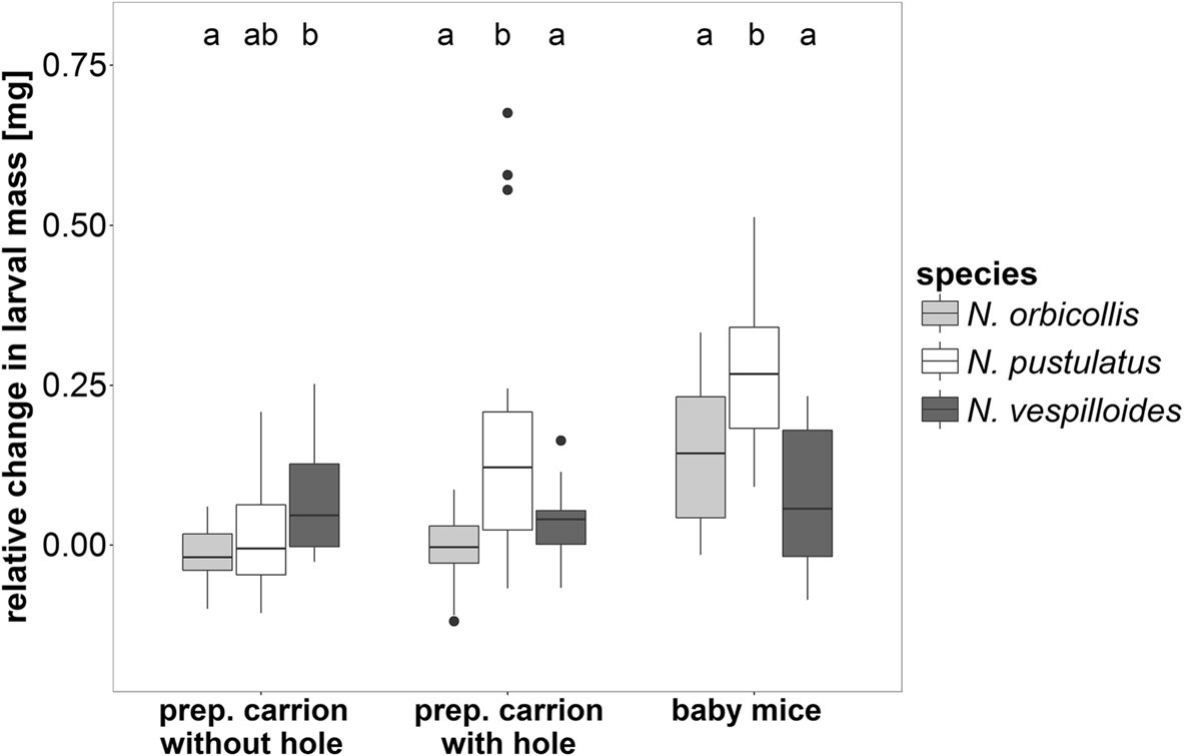
The change in larval mass from hatching to two hours after hatching in *N. orbicollis*, *N. pustulatus*, and *N. vespilloides* on different food sources [mg]. *N* = 15 for each species and treatment. Boxplots show median, interquartile range, minimum/maximum range. The dots are values that fall outside the interquartile range (> 1.5× interquartile range). Different letters indicate significant differences between species within one treatment

When we provided individual larvae with one of three different food resources, we found significant effects of treatment and species as well as a significant interaction on the relative change in larval mass (Table 1, see Fig. 2). Across all treatments, highly dependent *N. orbicollis* larvae gained less larval mass (mean 0.04 ± SE 0.02 mg) than independent *N. pustulatus* (mean 0.16 ± SE 0.03 mg) (Pairwise test: *P* < 0.001). There was no difference in the change in larval mass between *N. orbicollis* and *N. vespilloides* (mean 0.06 ± SE 0.01 mg) (Pairwise test: *P* = 1.00). The gain in larval mass was higher in *N. pustulatus* than in *N. vespilloides* (Pairwise test: *P* = 0.003). The type of food had an effect on change in larval mass, but this effect differed among the species (Table 1, see Fig. 2). *N. orbicollis* and *N. pustulatus* exhibited increased mass when provided with baby mice, whereas larval *N. vespilloides* did not. Also, cutting a hole into the carrion had a clear positive effect on weight increase in larval *N. pustulatus*, but not in the other two species.

**Table 1:** Results of the GLM of the effect of species (*N. orbicollis*, *N. pustulatus*, *N. vespilloides*), treatment (prepared carrion without hole, prepared carrion with hole, baby mice, overall sample size = 135) and the interaction of species and treatment on relative change in larval mass

factor	relative change in larval mass		
	*df*	*F*	*P*
species	2	13.44	**< 0.001**
treatment	2	17.61	**< 0.001**
species x treatment	4	6.00	**< 0.001**

Significant *p*-values are typed in bold

**Fig. 3:**
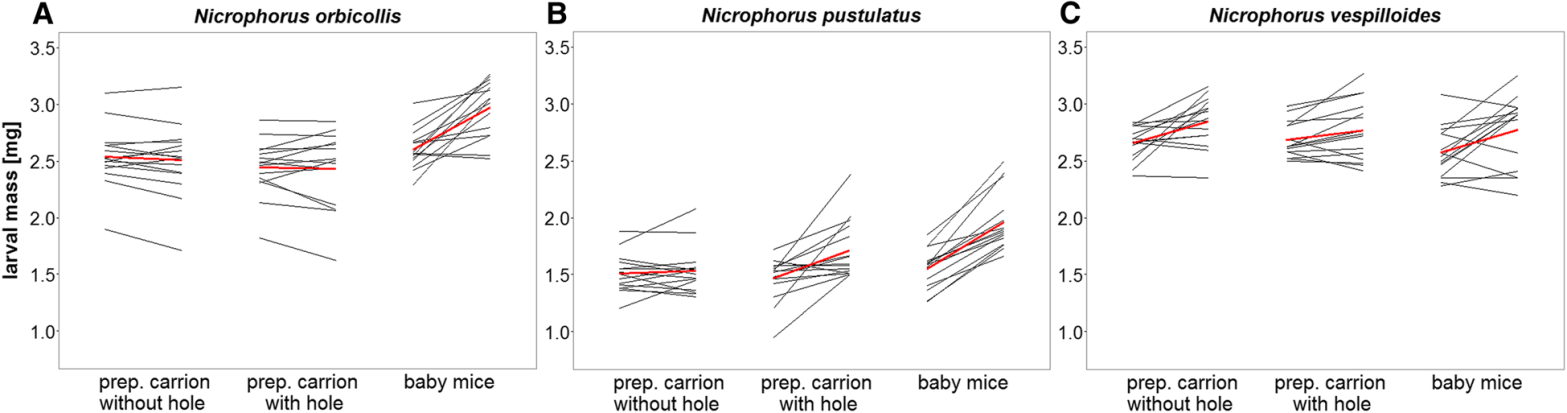
Larval masses [mg] at hatching (0 h) and after two hours on a specific food source in (**a**) *N. orbicollis*, (**b**) *N. pustulatus* and (**c**) *N. vespilloides*. N = 15 for each species and treatment. Each line represents one individual larva. The red line represents the mean of all larvae in one treatment

To better understand how the type of food affected the relative change in larval mass, we analysed each species separately. We found that the change in larval mass depended on the food provided in *N. orbicollis* and *N. pustulatus* (GLM with Gaussian errors: *F*_2,42_ = 20.52, *P* < 0.001 for *N. orbicollis*; *F*_2,42_ = 10.10, *P* < 0.001 for *N. pustulatus*), but not in *N. vespilloides* (GLM with Gaussian errors*: F*_2,42_ = 1.36, *P* = 0.27). In *N. orbicollis*, larvae only gained weight when provided with baby mice (Pairwise test: *P* < 0.001 for baby mice vs. prepared carrion with hole, baby mice vs. prepared carrion without hole, Fig. 3a). In contrast to highly dependent *N. orbicollis*, larvae of the more independent species were able to gain weight when provided with a carcass that was prepared by the parents. In *N. pustulatus*, larvae showed a greater increase in mass when provided with a prepared carcass with a hole (Pairwise test: *P* = 0.01) or baby mice than when provided with a prepared carcass without a hole (Pairwise test: *P* < 0.001, Fig. 3b). Larval *N. vespilloides* gained weight equally on the different types of food (see Fig. 3c).

Further, we should note that larval mass at hatching differed significantly among the three species (GLM with Gaussian errors: *F*_2,177_ = 517.69, *P* < 0.001). On average, larval *N. vespilloides* were heavier than larval *N. orbicollis* and larval *N. pustulatus* at hatching (Pairwise test: for both, *P* < 0.001). Larval *N. orbicollis* were, in turn, heavier than larval *N. pustulatus* (Pairwise test: *P* < 0.001).

### Experiment 3: Effect of oral secretions on larval *N. orbicollis*

**Fig. 4:**
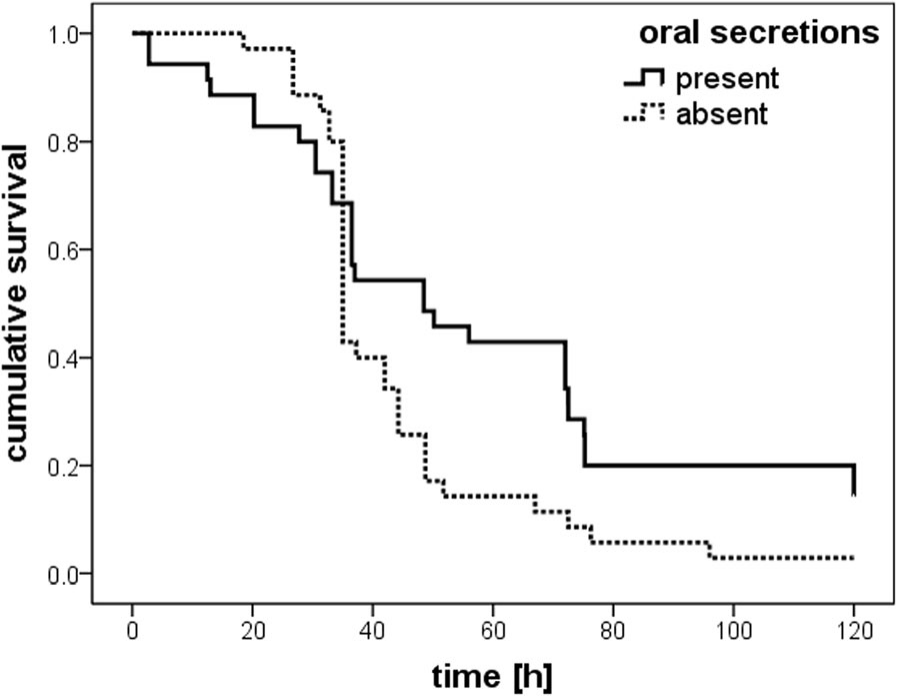
Survival of *N. orbicollis* larvae that received a paste of baby mice with or without oral secretions of parents [h]. *N* = 7 × 5 larvae for both treatments. Kaplan-Meier estimated survival curves

*N. orbicollis* larvae receiving baby mouse paste with oral secretions from parental beetles survived significantly longer than larvae that received plain baby mouse paste (log-rank test, *x*^*2*^ = 4.30, *P* = 0.038, see Fig. 4). On average, larvae that received baby mouse paste without oral secretions survived 43.84 (± 3.39) hours, whereas larvae receiving baby mouse paste mixed with oral secretions survived 58.27 (± 6.37) hours on average. Five out of 35 larvae fed baby mouse paste with oral secretions pupated, but only one of 35 larvae fed plain baby mouse paste did.

### Experiment 4: Duration of post-hatching care needed to ensure independence in larval *N. orbicollis*

**Fig. 5:**
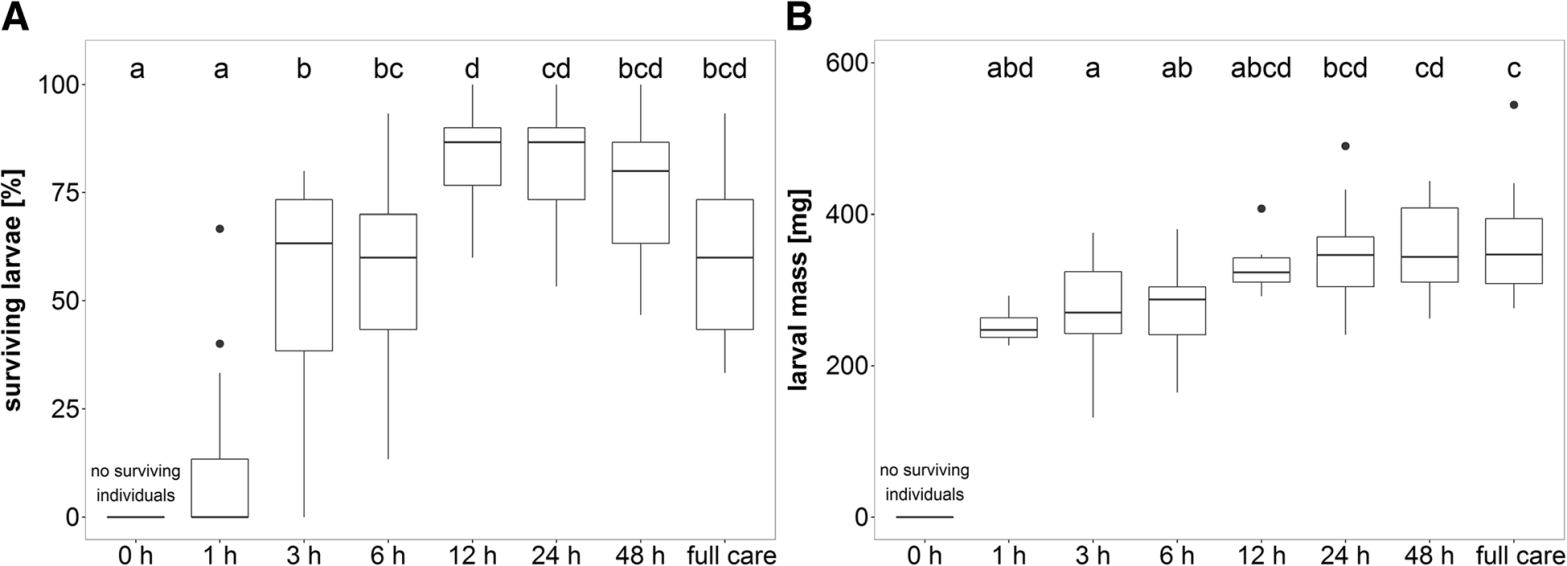
(**A**) Percent of larvae surviving to dispersal and (**B**) Mean larval mass per brood at dispersal [mg] of *N. orbicollis* larvae that received different durations of post-hatching care before parents were removed. N = 15 or 16 per treatment. Boxplots show median, interquartile range, minimum/maximum range. The dots are values that fall outside the interquartile range (> 1.5× interquartile range). Different letters indicate significant differences between treatments

Out of a total of 1815 *N. orbicollis* larvae, 951 survived. Larval survival was significantly affected by the duration of post-hatching care that parents provided (GLM with Poisson errors: *F*_7,113_ = 44.69, *P* < 0.001, see Fig. 5A). We found that 3 h of care significantly increased the number of larvae that survived to dispersal compared to broods that received 0 h (Pairwise test: *P* < 0.001) or 1 h of care (Pairwise test: *P* = 0.006). When considering the number of broods in which some larvae survived, 1 h of care is not yet sufficient to significantly increase survival rate (broods with/without surviving larvae: 0 h: 0/15; 1 h: 4/11; Fisher’s exact test: *P* = 0.100). However, it is clear that 3 h of care is sufficient to increase survival rate substantially; 87.5% of the broods had surviving larvae after only 3 h of parental attendance compared to none with 0 h of care (broods with/without surviving larvae: 0 h: 0/15; 3 h: 14/2; Fisher’s exact test: *P* < 0.001). In fact, 3 h of parental care did not differ from full care in terms of larval survival (Pairwise test: *P* = 1.00).

The duration of post-hatching care had a significant effect on larval mass (GLM with Gaussian errors: *F*_6,86_ = 6.31, *P* < 0.001, see Fig. 5B). The longer larvae received post-hatching care, the heavier they were when dispersing from the carcass. Within all surviving broods, larvae were significantly heavier when receiving 48 h of post-hatching care or full care than when receiving 3 h (Pairwise test: 48 h, *P* = 0.003; full care, *P* = 0.002) or 6 h of care (Pairwise test: 48 h, *P* = 0.009; full care, *P* = 0.005). As no larvae survived in the pre-hatching care treatment (0 h), we could not include this treatment in this analysis.

## Discussion

The results of our study reveal new insights into the proximate causes of the extreme dependence of *N. orbicollis* offspring on parental care, and the variation in offspring dependence among species. We found that starvation tolerance of larvae varied among species, but did not appear to be related to dependence on parental care. Newly hatched *N. orbicollis* were generally able to self-feed, but the capacity for utilizing different types of food was more limited than in the more independent species, *N. pustulatus* and *N. vespilloides*. Dependent *N. orbicollis* gained less weight when self-feeding than nutritionally independent *N. pustulatus*. In addition, our study revealed that even a highly processed liquefied carrion meal is not sufficient to secure larval survival in *N. orbicollis*; however, oral secretions of parents mixed into a purée of baby mice prolong the survival of larval *N. orbicollis* without parents, but not long enough for most larvae to pupate. Finally, we revealed that three hours of post-hatching care was sufficient to achieve a significant increase in the survival and final mass of the larvae of the most dependent species, *N. orbicollis*. Our results highlight key characteristics of offspring and parental traits that augment our understanding of offspring dependence on parental care. Below, we elaborate on the wider implications of these results.

The results of our first experiment make it unlikely that starvation tolerance is related to high levels of offspring dependence, but is instead more likely associated with variation in growth rate. Here, we tested whether larvae of the three species, *N. orbicollis*, *N. pustulatus*, and *N. vespilloides*, differ in their tolerance to starvation in the absence of parents. Combined with information on egg investment, represented by mass at hatching (see Fig. 3), the level of starvation tolerance could provide information on whether larvae are fast or slow metabolisers, or on the parental investment in egg composition, and could thus be related to the marked offspring dependence on parental provisioning in *N. orbicollis*. As expected, we found that offspring of the more independent species, *N. pustulatus*, were most tolerant to starvation and survived the longest in the absence of food. Surprisingly, however, the highly dependent larvae of *N. orbicollis* starved to death later than larvae of *N. vespilloides*, which show an intermediate dependence on parental care [25]. Here, hatchlings of *N. vespilloides* were the heaviest, followed by hatchlings of *N. orbicollis*, and then *N. pustulatus*, the lightest of the three species (but see [42]). Given their low mass at hatching, it is even more striking that most of the larval *N. pustulatus* were still alive when larvae of the other two species had all starved to death, suggesting that *N. pustulatus* are slow metabolisers. Generally, starvation resistance tends to increase with body size and larger energy stores, despite the greater absolute energy needs of larger individuals [43]. However, larval *N. vespilloides* are not only the heaviest at hatching, but also have the highest growth rate of the three species during the first 48 h with full care [25]. Faster growth rates are usually associated with a greater need for food and higher metabolic rates, making fast-growing individuals, such as *N. vespilloides*, more vulnerable to starvation when resources are limited [44].

The aim of our second experiment was to investigate whether hatchlings of *N. orbicollis* are able to self-feed, or whether traits necessary for self-feeding only develop at a later larval stage compared to the more independent species, which might explain strong offspring dependency on parental care. Larvae of passalid beetles, for example, differ in their ability to feed themselves and to construct feeding tunnels, and in their dependency on parental care [14]. Here, we found that newly hatched larvae of *N. orbicollis* were generally able to self-feed and gain weight when reared on baby mice, but not on prepared carcasses that parents usually use as a food resource for their offspring in nature. In contrast, no clear pattern was found in the two more independent species as larvae also increased in mass when provided with parent-prepared carcasses. It might not be surprising that larvae gain more weight on pieces of baby mice than, for example, on prepared carrion. First, baby mice are younger and probably have a higher water content, but fewer hard body parts than adult mice, making them more easily accessible for larvae. Second, pieces of baby mice are certainly fresher than the parent-prepared carrion. Further, the larvae of different species obviously differ in their ability to access and process different types of vertebrate carrion, which could be related to quantitative or qualitative differences in the oral digestive enzymes of larvae. It may be that the digestive system of young *N. orbicollis* hatchlings has evolved to rely more on pre-digested food from parents at the beginning, and later on, the slightly older larvae become able to consume and assimilate solid food on their own.

Alternatively, larval ability to self-feed might depend on species-specific characteristics of the mandibles. It is conceivable that mouthparts of *N. orbicollis* larvae may develop and sclerotize at a slower rate than the mouthparts of the other two species in our study, resulting in less robust mandibles that do not allow larvae to self-feed initially. Pukowski [27] observed that hatchlings and recently moulted larvae of *N. vespillo* are unable to self-feed, and ascribed this to their unsclerotized mouthparts. Only after five to six hours, were larvae observed to self-feed [27]. Thus, differences in the sclerotization rate of mandibles could contribute to the variation in self-feeding and offspring dependence on parental care. In species with obligatory parental care, such as *N. orbicollis*, selection on mandible sclerotization rate or other traits, such as the production of digestive enzymes that could facilitate nutritional independence of offspring, may be relaxed as parents assume a greater share of the services related to food intake. As the expression and maintenance of these traits is generally costly [45], traits related to self-feeding may only be expressed later in life when parents withdraw from providing parental care and offspring need to become independent. Generally, as soon as offspring traits are no longer in use because parents take over the tasks that secure offspring survival by providing parental care, a reduction in the relevant offspring traits is expected. This reduction, in turn, further drives the evolution of increased offspring dependency on parental care. For example, first instar neonates of wood-feeding *Cryptocercus* cockroaches, which exhibit elaborate biparental care, completely lack eyes and have a pale and thin cuticle [16]. The hindgut symbionts that help larvae to metabolise and digest wood are not fully established until the third larval instar [46]. Consequently, until that time, nymphs depend on their parents for nutrition and symbiont transfer [16]. Like *Cryptocercus*, first instar larvae of wood-feeding *Salganea* have a pale and transparent cuticle, and their eyes are present, but considerably reduced [16]. Larvae feed on parental oral fluids and are somewhat less dependent than larval *Cryptocercus*, but more dependent than *Panesthia* neonates that are well developed and show no interactions with parents [16]. In these three genera, the developmental characteristics of neonates appear to parallel a gradient of dependence on parental care [16].

Eggert et al. [28] showed that 12 h of parental care resulted in a significant increase in survival and growth of larval *N. vespilloides*, suggesting that this was due, in part, to the opening in the carcass that is created by the parents, thereby facilitating easier access of the larvae to the carrion. In an experimental evolution study, larvae descended from beetles reared in the absence of post-hatching care became increasingly independent, a result that was attributed to the ability of larvae to self-feed more efficiently or through morphological adaptation of larval mouthparts [47]. Although these behavioural or morphological adaptations are undoubtedly advantageous, their absence in larval *N. orbicollis* alone cannot explain their nutritional dependency. In our study, even an opening in the integument of a prepared carcass did not increase the efficiency of larvae to self-feed. Also, although larval *N. orbicollis* were able to consume small pieces of juvenile mouse carcasses, none of the larvae were able to survive more than 24 h in the absence of parents (A. Capodeanu-Nägler, pers. obs.). Even when provided with liquefied mouse carrion, most of the larvae did not survive to pupation.

One other factor that could account for the differences in self-feeding is the behaviour of larvae towards food when parents are absent. From a study on *N. vespilloides*, we know that larvae cooperate to penetrate the carcass when parents are absent [48]. One precondition for cooperation between siblings is that larvae need to aggregate first. Generally, larvae seem to be attracted to one another and without another larva, larvae of the more independent species may have directly attempted to feed. However, larvae of *N. orbicollis* that benefit most from their parents’ care, might be selected to focus on approaching their parents instead of converging to other larvae. Thus, especially when carcass preparation indicates the presence of parents by parent-derived cues on the carcass surface, larvae might wander around and search for a parent instead of attempting to feed (A. Capodeanu-Nägler, pers. obs.). Nevertheless, behavioural observations are needed to confirm these predictions.

Having shown that highly dependent *N. orbicollis* larvae are able to self-feed and increase in weight when provided with small pieces of baby mice, we attempted to determine whether they could be successfully reared in the absence of parents on a diet of homogenized mouse carrion mixed with oral secretions from parental beetles. We found that larvae reared on this diet were more likely to survive to dispersal than larvae receiving the same diet but without parental secretions. Thus, oral secretions of parents are clearly beneficial to *N. orbicollis* larvae, and may contain important symbionts, antimicrobial compounds, enzymes, or hormones. Eggert et al. [28] examined the importance of symbiont transfer in *N. vespilloides*, but found that the positive effects of parental provisioning on larval survival and growth were not mediated by the transfer of symbionts. However, the transfer of symbionts in *N. orbicollis* may be more important as larvae in this species are more dependent on parental provisioning. In addition, the beetles’ anal and oral secretions contain a wide range of compounds, some of which have antimicrobial properties [49–51], and express a variety of immune-related genes [52] with several antimicrobial peptides and lysozymes that are specifically upregulated in the presence of carrion [53, 54], and that could enhance offspring survival. Finally, parents may transfer growth-regulatory proteins or hormones that are essential for survival and development of dependent offspring. Juvenile hormone III (JH III), for example, has recently been found to be transferred to larvae by trophallaxis in ants [20]. In burying beetles, JH III plays a regulatory role in a multiple contexts [55–61], and parents might thus transfer some JH III when they regurgitate to larvae, which may contribute to their survival and growth (but see [62]).

Alternatively, oral secretions might signal the presence of parents to offspring. Carpenter ants, for instance, have been shown to exchange chemical signals by trophallaxis that help them to recognize nestmates [20, 21]. Likewise, oral secretions of burying beetles might have a signalling function that helps larvae to localize pre-digested food or initiates larval feeding. Nonetheless, despite receiving homogenized carrion mixed with oral secretions of parents, most of the larvae of *N. orbicollis* did not survive until dispersal. However, since we do not know the actual volume of oral fluids that parents transfer to larvae, we may have provided larvae with less than the requisite amount of oral secretions. In our last experiment, we showed that larval survival and mass of *N. orbicollis* increased with the duration of post-hatching care, which is not surprising as parental care usually enhances offspring fitness [7, 8]. More surprisingly, we found that survival of the highly dependent *N. orbicollis* larvae was significantly enhanced after only three hours of parental care. Why might such a short period of care have such a profound effect on offspring survival? For *N. vespilloides*, larval begging as well as parental provisioning is known to peak 24 h after hatching [32, 63]. However, we observed larvae begging and parents provisioning in the first three hours after hatching (A. Capodeanu-Nägler and M. Prang, pers. obs.). Thus, parents might provide begging larvae with enough food during these first few hours that larvae have sufficient energy to survive until they are efficient self-feeders.

In the light of the other results of our study, however, we find it more likely that the transfer of oral secretions and maybe also anal secretions might be crucial for larval survival and growth, especially in the first few hours after larval hatching. For example, if larvae are given a single dose of symbionts by the parents in the first three hours after hatching, they may be able to survive thereafter. Burying beetles are known to harbour a diverse gut microbiome including various *Yarrowia*-like yeasts [64]. *Yarrowia* are present in both adult and larval life stages, and are possibly involved in carrion digestion and preservation [65]. More recent studies have shown that burying beetle parents not only transfer microorganisms to larvae via oral secretions, but tightly regulate the microbiome of the carcass by applying anal and oral secretions to it, which serves not only as a nutritional resource, but also facilitates the vertical transmission of symbiotic microbiota to larvae [66–68]. Thus, the transfer of preservation- and digestion-related microbiota to the carcass during the first hours might enhance larval survival for the more dependent offspring of *N. orbicollis* after parents have been removed.

## Conclusions

Our study offers new insights into offspring and parental traits that appear to be relevant to the evolution of marked offspring dependence of certain species. We showed that tolerance to starvation differs greatly between species, but this is not likely to be associated with the high degree of offspring dependence in *N. orbicollis*. Nevertheless, *N. orbicollis* larvae are generally able to self-feed, but they are less efficient than larvae of the two more independent species. The variation in the efficiency to self-feed is probably not only due to differences in the structure or strength of larval mandibles, as larval *N. orbicollis* do not even survive when provided with liquefied mouse carrion for which the use of mandibles is redundant. As even short periods of parental care and easily accessible food containing oral secretions of parents significantly enhance survival of highly dependent *N. orbicollis*, we conclude that parental fluids must contain symbionts or other components that are crucial for larval survival. Thus, future studies should investigate the transfer and contents of oral fluids from parents to offspring more closely, which will further help to understand how coevolution drives an increasingly tight integration of offspring development and parental care [26, 69–73].

## References

[1] Behmer ST (2009). Insect herbivore nutrient regulation. Annu Rev Entomol.

[2] Slansky F (1982). Insect nutrition: an adaptationist's perspective. Fla Entomol.

[3] Bairlein F, Adams N, Slotow RH (1999). Energy and nutrient utilization efficiencies in birds–a review. Proceedings of the 22nd international ornithological congress, Durban.

[4] Castro G, Stoyan N, Myers JP (1989). Assimilation efficiency in birds: a function of taxon or food type?. Comp Biochem Phys A.

[5] Karasov WH (1990). Digestion in birds: chemical and physiological determinants and ecological implications. Stud Avian Biol.

[6] Scriber JM, Slansky F (1981). The nutritional ecology of immature insects. Annu Rev Entomol.

[7] Balshine S. Patterns of parental care in vertebrates. In: Royle NJ, Smiseth PT, Kölliker M, editors. The evolution of parental care. Oxford: Oxford University Press; 2012. p. 62–80.

[8] Clutton-Brock TH (1991). The evolution of parental care.

[9] Ballard O, Morrow AL (2013). Human milk composition: nutrients and bioactive factors. Pediatr Clin N Am.

[10] Shetty S, Bharathi L, Shenoy KB, Hegde SN (1992). Biochemical properties of pigeon milk and its effect on growth. J Comp Physiol B.

[11] Engberg RM, Kaspers B, Schranner I, Kösters J, Lösch U (1992). Quantification of the immunoglobulin classes IgG and IgA in the young and adult pigeon (*Columba livia*). Avian Pathol.

[12] Eraud C, Dorie A, Jacquet A, Faivre B (2008). The crop milk: a potential new route for carotenoid-mediated parental effects. J Avian Biol.

[13] Royle NJ, Russell AF, Wilson AJ (2014). The evolution of flexible parenting. Science.

[14] Tallamy DW, Wood TK (1986). Convergence patterns in subsocial insects. Annu Rev Entomol.

[15] Wong JWY, Meunier J, Kölliker M (2013). The evolution of parental care in insects: the roles of ecology, life history and the social environment. Ecol Entomol..

[16] Nalepa CA, Maekawa K, Shimada K, Saito Y, Arellano C, Matsumoto T (2008). Altricial development in subsocial wood-feeding cockroaches. Zool Sci.

[17] Costa JT (2006). The other insect societies.

[18] Maekawa K, Matsumoto T, Nalepa CA (2008). Social biology of the wood-feeding cockroach genus *Salganea* (Dictyoptera, Blaberidae, Panesthiinae): did ovoviviparity prevent the evolution of eusociality in the lineage?. Insect Soc.

[19] Shimada K, Maekawa K (2011). Description of the basic features of parent-offspring stomodeal trophallaxis in the subsocial wood-feeding cockroach *Salganea esakii* (Dictyoptera, Blaberidae, Panesthiinae). Entomol Sci.

[20] LeBoeuf AC (2017). Trophallaxis. Curr Biol.

[21] LeBoeuf AC, Waridel P, Brent CS, Gonçalves AN, Menin L, Ortiz D (2016). Oral transfer of chemical cues, growth proteins and hormones in social insects. elife.

[22] Nalepa CA, Bell WJ, Choe JC, Crespi BJ (1997). Postovulation parental investment and parental care in cockroaches. The evolution of social behavior in insects and arachnids.

[23] Byrd JH, Castner JL (2009). Forensic entomology: the utility of arthropods in legal investigations.

[24] Trumbo ST (1992). Monogamy to communal breeding: exploitation of a broad resource base by burying beetles (*Nicrophorus*). Ecol Entomol.

[25] Capodeanu-Nägler A, Keppner EM, Vogel H, Ayasse M, Eggert A-K, Sakaluk SK, Steiger S (2016). From facultative to obligatory parental care: interspecific variation in offspring dependency on post-hatching care in burying beetles. Sci Rep.

[26] Gardner A, Smiseth PT (2011). Evolution of parental care driven by mutual reinforcement of parental food provisioning and sibling competition. Proc R Soc B.

[27] Pukowski E (1933). Ökologische Untersuchungen an *Necrophorus* F. Z Morphol Ökol Tiere.

[28] Eggert A-K, Reinking M, Müller JK (1998). Parental care improves offspring survival and growth in burying beetles. Anim Behav.

[29] Scott MP (1998). The ecology and behavior of burying beetles. Annu Rev Entomol.

[30] Eggert A-K, Müller JK, Choe JC, Crespi BJ (1997). Biparental care and social evolution in burying beetles: lessons from the larder. The evolution of social behavior in insects and arachnids.

[31] Smiseth PT, Moore AJ (2002). Does resource availability affect offspring begging and parental provisioning in a partially begging species?. Anim Behav.

[32] Smiseth PT, Darwell CT, Moore AJ (2003). Partial begging: an empirical model for the early evolution of offspring signalling. Proc R Soc Lond B.

[33] Sikes DS, Venables C (2013). Molecular phylogeny of the burying beetles (Coleoptera: Silphidae: Nicrophorinae). Mol Phylogenet Evol.

[34] Anduaga S, Huerta C (2001). Effect of parental care on the duration of larval development and offspring survival in *Nicrophorus mexicanus* Matthews (Coleoptera: Silphidae). Coleopts Bull.

[35] Satou A, Nisimura T, Numata H (2001). Cost and necessity of parental care in the burying beetle *Nicrophorus quadripunctatus*. Zool Sci.

[36] Fetherston IA, Scott MP, Traniello JFA. Parental care in burying beetles: the organization of male and female brood-care behavior. Ethology. 1990;85:177–90.

[37] Scott MP, Traniello JFA (1990). Behavioural and ecological correlates of male and female parental care and reproductive success in burying beetles (*Nicrophorus* spp.). Anim Behav.

[38] Rauter CM, Moore AJ. Do honest signalling models of offspring solicitation apply to insects? Proc R Soc B. 1999;266:1691–6.

[39] Arce AN, Johnston PR, Smiseth PT, Rozen DE (2012). Mechanisms and fitness effects of antibacterial defences in a carrion beetle. J Evol Biol.

[40] Rauter CM, Moore AJ (2004). Time constraints and trade-offs among parental care behaviours: effects of brood size, sex and loss of mate. Anim Behav.

[41] Müller JK, Eggert A-K (1990). Time-dependent shifts between infanticidal and parental behavior in female burying beetles a mechanism of indirect mother-offspring recognition. Behav Ecol Sociobiol.

[42] Capodeanu-Nägler A, Eggert A-K, Vogel H, Sakaluk SK, Steiger S (2018). Species divergence in offspring begging and parental provisioning is linked to nutritional dependency. Behav Ecol.

[43] Stockhoff BA (1991). Starvation resistance of gypsy moth, *Lymantria dispar* (L.)(Lepidoptera: Lymantriidae): tradeoffs among growth, body size, and survival. Oecologia.

[44] Blanckenhorn WU (2000). The evolution of body size: what keeps organisms small?. Q Rev Biol.

[45] Lahti DC, Johnson NA, Ajie BC, Otto SP, Hendry AP, Blumstein DT (2009). Relaxed selection in the wild. Trends Ecol Evol.

[46] Nalepa CA (1990). Early development of nymphs and establishment of hindgut symbiosis in *Cryptocercus punctulatus* (Dictyoptera: Cryptocercidae). Ann Entomol Soc Am.

[47] Schrader M, Jarrett BJM, Kilner RM (2015). Using experimental evolution to study adaptations for life within the family. Am Nat.

[48] Schrader M, Jarrett BJM, Kilner RM (2015). Parental care masks a density-dependent shift from cooperation to competition among burying beetle larvae. Evolution.

[49] Degenkolb T, Düring R, Vilcinskas A (2011). Secondary metabolites released by the burying beetle *Nicrophorus vespilloides*: chemical analyses and possible ecological functions. J Chem Ecol.

[50] Hall CL, Wadsworth NK, Howard DR, Jennings EM, Farrell LD, Magnuson TS, Smith RJ (2011). Inhibition of microorganisms on a carrion breeding resource: the antimicrobial peptide activity of burying beetle (Coleoptera: Silphidae) oral and anal secretions. Environ Entomol.

[51] Arce AN, Smiseth PT, Rozen DE (2013). Antimicrobial secretions and social immunity in larval burying beetles, *Nicrophorus vespilloides*. Anim Behav.

[52] Vogel H, Badapanda C, Vilcinskas A (2011). Identification of immunity-related genes in the burying beetle *Nicrophorus vespilloides* by suppression subtractive hybridization. Insect Mol Biol.

[53] Palmer WJ, Duarte A, Schrader M, Day JP, Kilner RM, Jiggins FM. A gene associated with social immunity in the burying beetle *Nicrophorus vespilloides*. Proc R Soc B. 2016;283:20152733. 10.1098/rspb.2015.2733 PMC4795035 26817769

[54] Jacobs CGC, Steiger S, Heckel DG, Wielsch N, Vilcinskas A, Vogel H (2016). Sex, offspring and carcass determine antimicrobial peptide expression in the burying beetle. Sci Rep.

[55] Cotter SC, Kilner RM (2010). Sexual division of antibacterial resource defence in breeding burying beetles, *Nicrophorus vespilloides*. J Anim Ecol.

[56] Steiger S, Gershman SN, Pettinger AM, Eggert A-K, Sakaluk SK (2011). Sex differences in immunity and rapid upregulation of immune defence during parental care in the burying beetle, *Nicrophorus orbicollis*. Funct Ecol.

[57] Trumbo ST, Borst DW, Robinson GE (1995). Rapid elevation of juvenile hormone titer during behavioral assessment of the breeding resource by the burying beetle, *Nicrophorus orbicollis*. J Insect Physiol.

[58] Trumbo ST (1997). Juvenile hormone-mediated reproduction in burying beetles: from behavior to physiology. Arch Insect Biochem.

[59] Scott MP, Panaitof SC (2004). Social stimuli affect juvenile hormone during breeding in biparental burying beetles (Silphidae: *Nicrophorus*). Horm Behav.

[60] Panaitof SC, Scott MP, Borst DW (2004). Plasticity in juvenile hormone in male burying beetles during breeding: physiological consequences of the loss of a mate. J Insect Physiol.

[61] Engel KC, Stökl J, Schweizer R, Vogel H, Ayasse M, Ruther J, Steiger S (2016). A hormone-related female anti-aphrodisiac signals temporary infertility and causes sexual abstinence to synchronize parental care. Nat Commun.

[62] Crook TC, Flatt T, Smiseth PT (2008). Hormonal modulation of larval begging and growth in the burying beetle *Nicrophorus vespilloides*. Anim Behav.

[63] Smiseth PT, Lennox L, Moore AJ (2007). Interaction between parental care and sibling competition: parents enhance offspring growth and exacerbate sibling competition. Evolution.

[64] Kaltenpoth M, Steiger S (2014). Unearthing carrion beetles' microbiome: characterization of bacterial and fungal hindgut communities across the Silphidae. Mol Ecol.

[65] Vogel H, Shukla SP, Engl T, Weiss B, Fischer R, Steiger S (2017). The digestive and defensive basis of carcass utilization by the burying beetle and its microbiota. Nat Commun.

[66] Shukla SP, Vogel H, Heckel DG, Vilcinskas A, Kaltenpoth M (2017). Burying beetles regulate the microbiome of carcasses and use it to transmit a core microbiota to their offspring. Mol Ecol.

[67] Wang Y, Rozen DE (2017). Gut microbiota colonization and transmission in the burying beetle *Nicrophorus vespilloides* throughout development. Appl Environ Microb.

[68] Wang Y, Rozen DE (2018). Gut microbiota in the burying beetle, *Nicrophorus vespilloides*, provide colonization resistance against larval bacterial pathogens. Ecol Evol.

[69] Kramer J, Meunier J. The evolution of social life in family groups. bioRxiv. 2017:221192.

[70] Kölliker M, Royle NJ, Smiseth PT, Royle NJ, Smiseth PT, Kölliker M (2012). Parent—offspring co-adaptation. The evolution of parental care.

[71] Royle NJ, Alonzo SH, Moore AJ (2016). Co-evolution, conflict and complexity: what have we learned about the evolution of parental care behaviours?. Curr Opin Behav Sci.

[72] Badyaev AV, Uller T (2009). Parental effects in ecology and evolution: mechanisms, processes and implications. Philos T R Soc B.

[73] Uller T, Royle NJ, Smiseth PT, Kölliker M (2012). Parental effects in development and evolution. The evolution of parental care.

